# Machine Learning‐Enabled Tactile Sensor Design for Dynamic Touch Decoding

**DOI:** 10.1002/advs.202303949

**Published:** 2023-09-22

**Authors:** Yuyao Lu, Depeng Kong, Geng Yang, Ruohan Wang, Gaoyang Pang, Huayu Luo, Huayong Yang, Kaichen Xu

**Affiliations:** ^1^ State Key Laboratory of Fluid Power and Mechatronic Systems School of Mechanical Engineering Zhejiang University Hangzhou 310027 China; ^2^ Zhejiang Key Laboratory of Intelligent Operation and Maintenance Robot Hangzhou 310000 China; ^3^ School of Electrical and Information Engineering The University of Sydney Sydney NSW 2006 Australia

**Keywords:** human–machine interactions, laser‐induced graphene, machine learning, tactile sensor, touch decoding

## Abstract

Skin‐like flexible sensors play vital roles in healthcare and human–machine interactions. However, general goals focus on pursuing intrinsic static and dynamic performance of skin‐like sensors themselves accompanied with diverse trial‐and‐error attempts. Such a forward strategy almost isolates the design of sensors from resulting applications. Here, a machine learning (ML)‐guided design of flexible tactile sensor system is reported, enabling a high classification accuracy (≈99.58%) of tactile perception in six dynamic touch modalities. Different from the intuition‐driven sensor design, such ML‐guided performance optimization is realized by introducing a support vector machine‐based ML algorithm along with specific statistical criteria for fabrication parameters selection to excavate features deeply concealed in raw sensing data. This inverse design merges the statistical learning criteria into the design phase of sensing hardware, bridging the gap between the device structures and algorithms. Using the optimized tactile sensor, the high‐quality recognizable signals in handwriting applications are obtained. Besides, with the additional data processing, a robot hand assembled with the sensor is able to complete real‐time touch‐decoding of an 11‐digit braille phone number with high accuracy.

## Graphical abstract

A data‐driven strategy of a flexible triboelectric nanogenerator‐based tactile sensor and machine learning co‐designed system is proposed to optimize the classification accuracy of output signals from six contact modalities. Via extracting the subtle signals from the raw data by machine learning algorithms, this system is able to dynamically decode an 11‐digit multi‐point braille numbers.

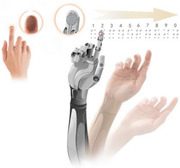

## 1 Introduction

To mimic the somatosensory systems of human beings, a variety of skin‐like sensors have been developed to track abundant stimuli, such as touch,^[^ ^1^ ^]^ pressure,^[^ ^2^ ^]^ vibration,^[^ ^3^ ^]^ temperature,^[^ ^4^, ^5^ ^]^ and humidity.^[^ ^6^ ^]^ In particular, tactile perceptions that imitate mechanoreceptors play crucial roles in shaping interactions with complex surroundings.^[^ ^7^, ^8^, ^9^, ^10^, ^11^ ^]^ This enables their wide applications in healthcare measurement, intelligent robots, human–machine–environment interfaces as well as augmented/virtual reality.^[^ ^12^, ^13^, ^14^ ^]^ In these applications, to enhance the reliability of signal recognition, machine learning （ML） algorithms are emerging as effective means to reveal correlations and subtle differences among multichannel datasets.^[^ ^15^, ^16^, ^17^ ^]^ For example, speech‐related or triboelectric‐based signals share pretty similar information, which has been coupled with machine learning algorithms, such as support vector machine (SVM), convolutional neural network (CNN), and rapid situation learning (RSL) to boost data identification.^[^ ^18^, ^19^, ^20^, ^21^ ^]^ This strategy has been applied to recognize speech, characterize gait, as well as control gestures.

To achieve a desirable sensor, general goals target at acquiring the high‐performance properties, such as superior sensitivity, wide working range, excellent repeatability， etc.^[^ ^22^ ^]^ This is typically accompanied with diverse trial‐and‐error attempts based on intuition or quasi‐random fabrication parameters.^[^ ^23^ ^]^ Specific applications are then performed using the optimal device with the decent computational analysis of data output. However, such a forward strategy almost isolates the design of sensors and resulting applications, which may cause increasing data burden, weaken the personalized signal features, and reduce the efficiency of computational analysis. In contrast, inverse design involves the statistical learning methodologies in the design phase of sensing hardware, which challenges the intuition‐driven sensor.^[^ ^24^, ^25^ ^]^ Such a data‐driven design approach links the training data with the target sensing information, which bridges the gap between the hardware (device structures) and software (algorithms).

In this work, a co‐designed flexible sensor system realized by an ML‐reinforced design strategy is proposed to optimize the output signals of laser‐induced graphene (LIG)‐based tactile sensors via signal quality enhancement under various evaluation criteria. Owing to the task‐oriented requirement in dynamic friction interactions between the sensor and measured objects, the discrimination of signals induced by six representative dynamic touch modalities is considered as a generalized evaluation criterion during the sensor design optimization. These criteria, based on SVM and several statistical standards, are applied to intelligibly disclose the quality of signals, enabling a high classification accuracy (≈99.58%) of tactile perception in six dynamic touch modalities. This co‐designed system is able to realize visual discrimination in handwriting signals and recognition of braille numbers.

## 2 Results and Discussion

**Figure 1:**
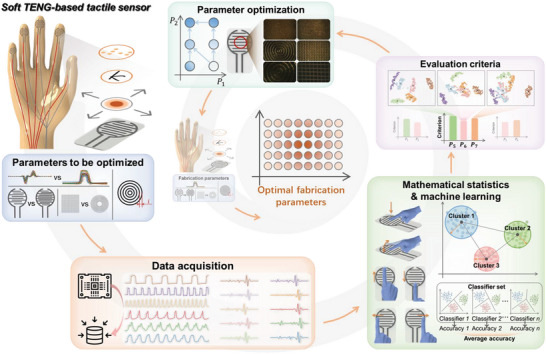
Schematic of the machine learning‐assisted sensor design via fabrication parameters optimization.

In the proposed ML‐assisted sensor design, a set of parameters related to triboelectric nanogenerator (TENG)‐based tactile sensing performance under contact‐sliding mode is selected as the initial optimization targets, including types of the readout signals (e.g., current and voltage), distribution of electrodes, shapes, and density of microstructures (**Figure** **1**). Six dynamic touch modalities are performed on the sensor by the finger skin, including press, pat, up, down, left, and right sliding. These multi‐directional interactions are selected for targeted tasks to evaluate each fabrication parameter. The signal qualities are evaluated by an SVM‐based ML algorithm and several statistical criteria to screen the optimal value for each fabrication parameter. The evaluation and optimization process are repeated until all targets are optimized, giving rise to a group of desirable parameters for sensor fabrication and data acquisition. Different from the general intuition‐driven sensor design, this work provides a data‐driven approach to optimizing fabrication parameters for the targeted applications, which closely connects the device design and algorithms.

**Figure 2:**
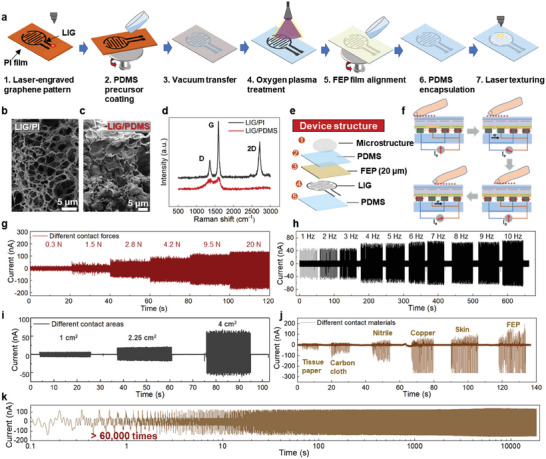
Characterizations of the TENG‐based tactile sensor. a) Fabrication procedures of the sensor. SEM of b) porous LIG and c) PDMS‐embedded LIG. d) Raman results of LIG on PI and LIG on PDMS after transfer. e) Explosive view of the device. f) Working mechanism of the sensor. The triboelectric current of TENG under stimuli of g) different contact forces, h) frequencies and i) contact areas. j) The triboelectric current induced by various contact materials including tissue paper, carbon cloth, nitrile, copper, skin, and FEP. k) The wear‐resisting property of the proposed triboelectric sensor under over 60 000 cycles of friction.

**Figure** **2**a illustrates fabrication processes of a flexible TENG‐based tactile sensor. First, the interdigital electrodes were fabricated via a digital infrared (IR) laser direct writing technique. By virtue of the laser‐induced thermal effect, LIG patterns could be obtained by laser carbonization of a polyimide (PI) film. The LIG method features rapid patterning without precursors as well as active materials’ transfer to soft elastomers.^[^ ^26^, ^27^, ^28^, ^29^, ^30^, ^31^, ^32^, ^33^ ^]^ Then, the LIG/PI film was spin‐coated with polydimethylsiloxane (PDMS). After the PDMS solution infiltrates into the porous LIG, a heat‐curing process was performed, followed by peeling off the PI to achieve a thin LIG/PDMS film. To introduce additional electronegative groups as well as remove the surface impurities, oxygen plasma treatment of LIG/PDMS was conducted. The next crucial process was the alignment of Fluorinated ethylene propylene (FEP) on LIG/PDMS, which aims to enhance the triboelectric effect during contact‐sliding processes. Finally, the device was encapsulated by a layer of PDMS. During the co‐designed process, the grid‐like and fingerprint‐like microstructures were created on the top layer of encapsulation PDMS by laser texturing.

To validate the properties of LIG before and after transfer, the surface morphology and Raman spectra of LIG/PI and LIG/PDMS were characterized, respectively. During the laser engraving process, the rapid release of gas leads to the high porosity of structures (Figure 2b). After the transfer of LIG onto PDMS, the porous structures are well preserved (Figure 2c). Furthermore, the LIG presented similar Raman peaks (i.e., D, G, and 2D peaks) to graphene at 1349, 1589, and 2711 cm^−1^, respectively (Figure 2d). However, the LIG/PDMS is observed with relatively weak intensity of D and G peaks, probably caused by the mechanical delamination to introduce additional defects.^[^ ^34^ ^]^

As illustrated in the fabrication steps, the TENG‐based tactile sensor is composed of four layers including top and bottom PDMS encapsulations, interdigital LIG electrodes and a FEP film (Figure 2e). When the finger touches the surface of PDMS, human skin is positively charged due to their different electron affinities. Based on the principle of electrostatic induction, the horizontal sliding of charged finger creates electron transfer with induced current between the two LIG electrodes (Figure 2f). In such a case, the interdigital design of LIG electrodes allows for a gradient electron transfer that the induced current alternates negatively and positively to balance the potential difference between the two electrodes. This mechanism effectively incorporates information related to dynamic touch modalities into the raw data. Notably, two conductive electrodes are applied for the free‐standing TENG rather than utilize the grounding method. Therefore, the accumulated charges of a moving object can be transported between the cross‐distributed interdigital electrodes for facile measurements.

To characterize the performance of this tactile sensor, open‐circuit voltage, and short‐circuit current were recorded as functions of contact forces, frequencies, areas, and materials. For contact forces, both open‐circuit voltage and short‐circuit current of TENG are observed with gradient increases as the force increases from 0.3 N to 20 N (Figure 2g; Figure S1a,b, Supporting Information). With the increase of contact frequencies, the short‐circuit current presents a positive correlation while the open‐circuit voltage negligibly changes (Figure S1c, Supporting Information). This proves that the output voltage of proposed TENG is independent of frequency. A minor degradation of short‐circuit current is captured at the frequency of 8 Hz probably due to the saturation of ionized charges (Figure 2h).

To enhance the performance of tactile sensor, the effect of contact area and dielectric properties of contact materials were investigated. Notably, both open‐circuit voltage and short‐circuit current evidently increase with contact area from 1–4 cm^2^ (Figure 2i; Figure S1d, Supporting Information). In addition, contact materials with high permittivity and triboelectric effect, such as copper, human skin, and FEP film, generate short‐circuit current with nearly three times higher (> 350 nA) than tissue paper (<50 nA), illustrating a more remarkable signal‐to‐noise ratio (Figure 2j). Since the tactile sensor was designed for feature signal extraction in a touch‐sliding process, a high signal‐to‐noise ratio plays a significant role in the data analysis. Based on the sensing principle, the small interferences (<7.5%) caused by both high moisture (>90% RH) and temperature variations (25–60 °C) on the output amplitude of this tactile sensor is almost negligible (Figure S2, Supporting Information). In this case, an FEP film was chosen as the middle layer material between the interdigital LIG electrodes and PDMS to further enlarge the contact signals. To validate the durability of this device, over 60 000 times of repeated slaps were conducted. The negligible change in performance reflects the stability and signal repeatability of TENG as a tactile sensor (Figure 2k).

**Figure 3:**
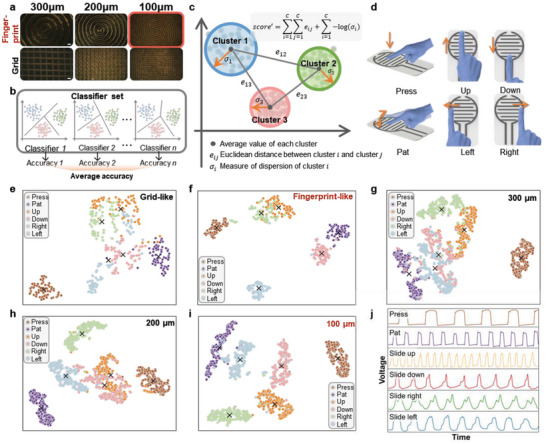
Optimization of fabrication parameters assisted by machine learning methods. a) Different shapes and sizes of microstructures on the top layer of PDMS surface. Scale bars: 100 µm. b) Calculation of the average classification accuracy by SVM classifiers. c) Calculation of the separability and dispersion of data after the dimensionality reduction. d) Schematic of six dynamic touch modalities. Dimensionality reduction results of the data collected from sensors with e) fingerprint‐like and f) grid‐like microstructures, respectively. Dimensionality reduction results of the data collected from sensors with microstructures of different sizes at g) 300, h) 200, and i) 100 µm, respectively. j) Output of the parameter‐optimized sensor to six touch modalities.

The design of TENG‐based flexible tactile sensor reinforced by ML involves several fabrication parameters, especially the arrangement of electrodes, shape, and density of surface microstructures (**Figure** **3**a). Signal qualities were assessed to quantify the effects of these parameters. It should be noted that criteria to evaluate signal quality should be as plain and general as possible for disclosing the properties of signals, as a sophisticated one may muffle them. This often happens along with an over‐fitting problem, where a strong criterion, such as neural network, reports high scores regardless of the signals’ quality. Thus, this study proposed two easily implemented criteria, i.e., the average classification accuracy by a set of SVMs and the separability of signals in a dimensionality‐reduced space, to select a group of optimal fabrication parameters. Given a set of categorized data, *N* SVM classifiers were implemented in parallel to classify them, during which an average classification accuracy $\frac{1}{N}\sum_{k\;=\;1}^{N}Acc_{k}$ was obtained as the first criterion (Figure 3b). Next, the t‐distributed stochastic neighbor embedding (t‐SNE) algorithm was employed to reduce the dimensionality of raw signals into a 2D space (range of its two dimensions were projected into [0,  1]), where the separability of signals was evaluated as diagrammed in Figure 3c. Specifically, the clustering center of each category was computed as the mean value of involved data, followed by the computation of Euclidean distance between each pair of centers as the next criterion (e.g., *e*_ij_ indicates the Euclidean distance between clustering center *i* and *j*). In addition, the dispersion of each cluster was approximated by the standard deviation σ_*i*_. The three indicators reflected the properties of collected signals, giving rise to the total criterion as:

(1) $$score\;=\frac{1}{N}\;\sum_{k\;=\;1}^{N}Acc_{k}+\sum_{i\;=\;1}^{C}\sum_{j\;=\;1}^{C}e_{ij}+\sum_{i\;=\;1}^{C}-log\left(\sigma_{i}\right)$$

where *N* is the number of SVM classifiers, *C* is the number of clustering centers. It should be noted that we performed a normalization technique on three indicators to project them into the range [0,  1], in order to eliminate the effect of dimensions. Additionally, since smaller deviation was expected for signals of higher repeatability, σ_*i*_ was modified as its negative logarithm form − logσ_*i*_. By maximizing the criterion *score* in Equation (1), the fabrication parameters can be optimized.

In order to generate the categorized data to support the proposed criteria, six dynamic touch modalities were performed on the tactile sensor to produce datasets for the optimization of fabrication parameters. The interdigital LIG electrodes can generate alternating signals based on the working mechanism of TENG. These signals exhibit distinct temporal features dominated by different sliding directions. Therefore, the six touch modalities are determined as press, pat, up, down, left, and right sliding. In the process of data collection, a semi‐automatic annotation strategy is proposed to segment and annotate the collected data as illustrated in Figure S3 (Supporting Information) and summarized in Algorithm S1 (Supporting Information), where an anchor sample is applied to search for the alternative data samples.

As the induced current and voltage are highly associated with the sensing performance of TENG‐based tactile sensor, both types of signal output are compared. First, the repeatability of signals is examined as shown in Figure S4a,b (Supporting Information), where voltage signals exhibit better repeatability with smaller deviations from the average signal (black dotted line in each subplot) while current signals are distributed irregularly and far from the cluster center. Further analysis in the two‐dimensional space (Figure S4c,d, Supporting Information) reveals that the voltage signals are better clustered with clearer boundaries among different touch modalities compared to the current signals. Besides, ten linear SVM classifiers were implemented to classify these data, resulting in an average accuracy of 94.278% and 95.579% for voltage and current signals, respectively. The separability and dispersion are then calculated using the criteria defined in the previous section, resulting in [3.636, 11.877] and [3.528, 11.267] for voltage and current signals, respectively. To eliminate the influence of different dimensions, we normalized the results by transforming [94.278%, 3.636, 11.877] and [95.579%, 3.528, 11.267] into [0, 1, 1] and, [1, 0, 0] respectively. This indicates that voltage signals receive a total criterion score of two, while current signals receive one. Therefore, voltage‐type signals are a better choice as the sensor's output in the proposed design.

Second, the distribution density of electrodes in the proposed design can affect the complexity of signals, which can be reflected by subtle peaks. To figure out the impact of electrode density on classification results, the tactile performance with sparse and dense distribution of electrodes is investigated. Clustering results in Figure S5 (Supporting Information) reveal that data from arrangements with sparse electrode pairs were more distinguishable among different classes. Scores for the sparse and dense arrangements are calculated, resulting in [96.975%, 3.695, 14.759] and [95.958%, 3.528, 11.542], respectively. This illustrates that a denser arrangement of electrodes allows more sensitive subtle peaks in the signals, but it also generates more superimposed peaks, leading to the difficulty in signal recognition by an ML algorithm. After normalization, the scores were [1, 1, 1] and [0, 0, 0], resulting in total criterion scores of three and zero, respectively. Therefore, the sparse arrangement of electrode pairs is selected in the proposed design.

After evaluating the types of output signals and the density of electrodes, the output comparison of surface microstructures with different shapes and intervals textured by laser is further investigated. Similar to the natural grooves on finger skin, rugged microstructures on top layer of PDMS are designed to reduce the disturbance of viscous effect on signal quality, resulting in the further enhancement of data discrimination. To validate it, two microstructures commonly applied on tactile sensors were studied, including fingerprint‐like and grid‐like shapes with different groove intervals (Figure 3a). Via comparing the voltage output of six touch modalities, Figure S6 (Supporting Information) illustrates that data from the fingerprint‐like structure show a concentrated distribution around the average values, while data from the grid‐like structure exhibit more instability. Besides, the results of dimensionality reduction shown in Figure 3e,f indicate that the data of the fingerprint‐like structure present a stronger separability with a much more concentrated distribution within classes, compared to that of the grid‐like structure. The calculated scores based on the proposed criteria for the fingerprint‐like and grid‐like structures are [96.50%, 4.227, 14.958] and [95.056%, 3.725, 11.783], respectively. After normalization, the scores are transformed into [1, 1, 1] and [0, 0, 0], respectively, indicating that the fingerprint‐like structure is more appropriate for the sensor.

Furthermore, the performance of fingerprint‐like microstructure is found to be affected by the interval between adjacent grooves (Figure S7a, Supporting Information). During the co‐design process, two sensors are first fabricated with groove intervals of 200 and 300 µm, respectively and the data corresponding to six touch modalities are collected. The proposed criteria are used to calculate their scores, resulting in [93.533%, 3.581, 12.670] and [94.558%, 3.561, 11.283] for 200 and 300 µm, respectively (Figure S7b,c, Supporting Information). Similar results are observed in Figure 3g,h. It suggests that a smaller interval between grooves leads to the better performance of sensor. Therefore, a fingerprint‐like pattern with a groove interval of 100 µm is produced on the sensor's surface, which almost reaches the processing limit of this IR laser system. Compared to sensors with the larger intervals of 200 and 300 µm, the sensor with a fingerprint‐like interval of 100 µm exhibits better repeatability, contributing to a more concentrated clustering within classes (Figure 3i; Figure S7d, Supporting Information). The clearer boundaries among different classes also indicate higher separability of its raw data. This sensor achieves scores of [98.033%, 4.044, 12.535]. After normalization, scores of [1, 0.902, 1], [0.042, 1, 0], and [0, 0, 0.228] are obtained for groove intervals of 100, 200, and 300 µm, respectively. As a result, the total scores are calculated as 2.902, 1.042, and 0.228, indicating that a groove interval of 100 µm is the optimal selection.

In summary, to enhance the performance of TENG‐based tactile sensor, a set of evaluation criteria is developed and applied to select the desired type of output signals and optimize the fabrication parameters. Comparison results are summarized in **Table** **1**. After evaluation, voltage‐based signals are selected as the sensor output. Meanwhile, sparsely distributed electrode pairs and fingerprint‐like microstructures with the smaller interval contribute to the enhancement of sensor performance. Figure 3j exhibits the output of sensor with the optimized parameters on six touch modalities, enabling a remarkable classification accuracy of 99.58% by a tuned linear SVM classifier.

**Table 1:** Summarization of the comparison results during the optimization process.

Fabrication parameters		Accuracy	Separability	Dispersion
Output signal	**Voltage**	94.278%	3.636	11.877
	Current	95.579%	3.528	11.267
Electrode arrangement	**Sparse**	96.975%	3.695	14.759
	Dense	95.958%	3.528	11.542
Microstructure shape	**Fingerprint‐like**	96.500%	4.227	14.958
	Grid‐like	95.056%	3.725	11.783
Microstructure density	**100 µm**	98.033%	4.044	12.535
	200 µm	93.533%	3.581	12.670
	300 µm	94.558%	3.561	11.283

**Figure 4:**
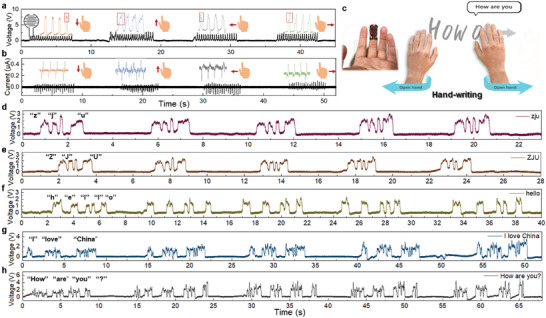
Optimized tactile sensing performance in handwriting applications. The outputs in the form of a) open circuit voltage and b) short circuit current of the tactile sensor by sliding a finger at four directions. Insets are the enlarged peaks extracted from the original results in corresponding contact directions. c) Schematic of handwriting demonstration by attaching the device on fingers. d–h) Handwriting results of different English words and sentences based on the proposed tactile sensor.

Benefiting from the high recognition accuracy of tactile sensor optimized by machine learning, the recognizable signal differences of finger sliding at four different directions on the device surface are observed (**Figure** **4**a,b). A critical feature of freestanding TENG is that the proximity of an arbitrarily charged object can easily cause a significant increase in open circuit voltage, while a continuous, weak variation of subtle signals is induced by the sliding process. Notably, these hidden peaks displayed in insets represent the dynamic friction process. Based on the aforementioned ML‐assisted selection of the readout signals, the open‐circuit voltage achieves higher scores for the separability and dispersion of data than that of short‐circuit current. Thus, the handwriting of different letters is identified under voltage measurement to realize character recognition (Figure 4c). Figure 4d–h present the real‐time recognition of English words and sentences using the sensor. For different words and sentences, it is easy to distinguish them by observing the signal features. However, for the same word in uppercase and lowercase, like “ZJU” and “zju”, it is a bit difficult to recognize them only relying on the voltage signals. Thus, to further optimize the capability of signal recognition, additional ML algorithms are required for computational analysis.

**Figure 5:**
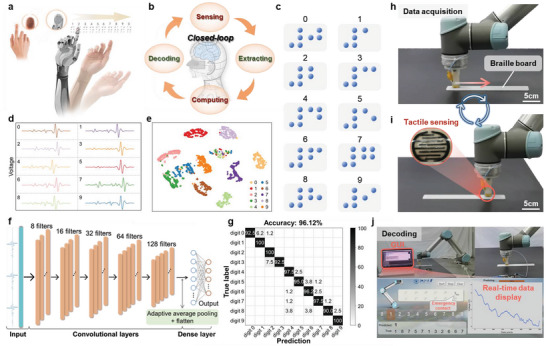
Dynamic decoding feedback of muiti‐point contact by assembling a tactile sensor on a robotic finger. a) Concept illustration of a recognition feedback system simulating finger perception. b) Close‐loop flow chart of a robotic sensing system that mimics human skin perception. c) Schematic of braille numbers from 0 to 9. d) Real‐time signals of ten braille numbers processed by a high‐pass filter. e) Dimensionality‐reduced clustering results of ten braille numbers. f) A 1D CNN model with 128 filters used for data classification. g) Classification results of ten braille numbers after 320 cycles of data acquisition. h,i) Real‐time data acquisition process of an 11‐digit braille phone number realized by a smart robotic finger. j) Real‐time signal feedback and display of recognition results of the braille phone number on a graphical user interface.

To mimic the superior tactile behavior of human skin, the proposed tactile sensor is integrated onto a robot finger for braille recognition (**Figure** **5**a). With the addition of ML algorithms, the robot‐sensor system works at a closed loop of sensing, extracting, computing, and decoding information (Figure 5b). Compared to the handwriting applications, braille recognition is more challenging due to the recognition mode shifts from a single‐point contact to a multi‐point contact in a dynamic process. Initially, touch signals of ten braille numbers are collected via the robot hand (Figure 5c). Through preprocessing by a high‐pass filter, the subtle signals induced by sliding process are extracted. In the process of data acquisition, great shape similarity in these braille numbers leads to the recognition difficulty of corresponding signals via visual discrimination (Figure 5d). This is also illustrated by the overlaps among clusters in the dimensionality‐reduced results (Figure 5e). Thus, a one‐dimensional CNN model is utilized to automatically perform feature learning and classification after data acquisition (≈320 times per number) (Figure 5f). Attributed to the powerful non‐linear fitting capability of CNN, an average classification accuracy of 96.12% is achieved (Figure 5g). As can be seen, most of the braille numbers achieve a classification accuracy over 95% (i.e., digit 1, 2, 4, 5, 6, 7, 9), while the lower accuracies are observed in the digit 0, 3, 8. This tiny performance difference probably results from the force control accuracy and repetition accuracy of the manipulator during data acquisition. Such phenomena may also affect the accuracy of online recognization of braille numbers, resulting in a classification accuracy of 93.33% in online decoding process (Video S1, Supporting Information).

An additional experiment was performed to evaluate the sensor's recognition accuracy before the removal and after reinstallation. The data collected before the detachment were synthesized as datasets $S_{\mathrm{train}}$ and $S_{\mathrm{test}}^{1}$, which were used to train and subsequently test the classifier, respectively. Another dataset denoted as $S_{\mathrm{test}}^{2}$ was collected after the sensor's detachment and reinstallation to evaluate the system comprising the tactile sensor and the pre‐trained classifier. As shown in Figure S8 (Supporting information), data collected from different Braille numbers are separate distinguishably while data of the same number are clustered together. This implies that the wearing variability does not change the sensor's capability of distinguishing different textures. Besides, a training accuracy of 99.25% for $S_{\mathrm{train}}$, a test accuracy of 96% for $S_{\mathrm{test}}^{1}$, and a test accuracy of 87.5% for $S_{\mathrm{test}}^{2}$ were achieved, respectively. Notably, a decline in classification accuracy is observed after the sensor's detachment and reinstallation. This is attributed to the alteration in data distribution, but this issue can be effectively addressed via just collecting a small scale of new data and fine‐tuning the classifier.

To validate the feasibility for practical applications, this smart robot hand equipped with the sensitive tactile sensor is applied to decode 11‐digit phone numbers in real time. After printing a group of braille phone numbers, the robot hand is programmed to continuously perceive each number and provide real‐time signal feedback, together with the display of recognition results on a graphical user interface (GUI) (Figure 5h,i). The GUI is designed for the number output after identification and output visualization (Figure 5j; Video S2, Supporting Information). With this capability, such the smart robotic hand is able to call emergency contact for people with disabilities as well as affords high potentials as prosthetics for rapid and accurate recognition just like human hands.

## 3 Conclusion

In summary, we introduced a TENG‐based flexible tactile sensor that enables ML‐assisted device design in output signal selection and fabrication parameter optimization. After setting the evaluation criteria, the parameter values of output signal, distribution density of electrodes as well as diverse surface carved microstructures are compared and selected according to the statistical analyses of six contact modalities. Based on the comprehensive evaluation of fabrication parameters and machine learning co‐designed tactile performance, the classification accuracy of ≈99.58% is obtained, which is higher than that before parameter optimization (≈95.579%). Given the optimal tactile sensing performance, the tactile sensor is successfully applied for handwriting recognition of various English letters and sentences. Furthermore, via applying a customized CNN model to extract features and estimate the decision boundaries, the classification accuracy of ten braille numbers is achieved at 96.12%. To imitate human perceptual feedback, a smart robot hand assembled with sensors accomplishes the task of dynamic identification of an 11‐digit braille phone number. This work provides guidances to purposively construct the sensor based on an inverse design strategy, which challenges the intuition‐driven sensor design.

## 4 Experimental Section

### Materials

Polyimide (PI) films (50 µm) were purchased from Dupont (USA). Fluorinated ethylene propylene (FEP) films were supplied by a company named Floroplastic Materials (China). Polydimethylsiloxane (PDMS) precursors and curing agents were obtained from Dow corning (sylgard 184, USA).

### Fabrication of LIG/PDMS‐Based Tactile Sensor

The interdigital LIG electrodes were fabricated by laser patterning (power: 7.24 J cm^−2^) on a PI substrate utilizing an infrared (IR) laser system at a wavelength of 10.6 µm. Then, the LIG/PI film was uniformly spin‐coated with PDMS precursors at a speed of 800 rpm min^−1^ for 2–3 times. After vacuum treatment and solidification at 90 °C for 30 min, the LIG patterns could be well transferred onto the PDMS surface (thickness: ≈180 µm). Next, the surface of LIG/PDMS was treated by oxygen plasma, followed by the alignment of a FEP film. Finally, the tactile sensor was obtained by encapsulating PDMS on the top of the FEP film.

### Characterizations

Surface morphology of porous LIG was characterized by a scanning electron microscope (SEM) (Hitachi, SU3500, Japan). A Raman spectrometer ((LabRAM Soleil, HORIBA, Japan) was applied to analyze the Raman spectra of LIG/PI and LIG/PDMS. A linear motor (DA60‐B1‐T60‐C010‐0.2, Dynamikwell Technology, China) was used to apply different stimuli for the characterization of triboelectric performance. The open circuit voltage and short circuit current were collected by an electrometer ((Keithley 6514, Tektronix, USA). For the data collection in two applications, the open circuit voltage was obtained via a portable Arduino board.

### Data Collection

Data collection was involved in two cases, including the ML‐guided sensor design and recognition of braille numbers. For the former, participants were instructed to perform six dynamic touch modalities (100 times each). An electrometer was used to measure and record the induced signals in the current/voltage form, followed by a band‐pass filter to reduce noise. As described in Figure S3 and Algorithm S1 (Supporting Information), an anchor sample for each touch modality was first selected, and a similarity matching algorithm was used to search for the remaining samples, resulting in 600 samples per class as a dataset. This process was repeated several times according to the iterations of fabrication parameter optimization. That is to say that an exclusive dataset was collected for each parameter. Considering that it was the data quality to be assessed, all the data in a dataset were used to train the SVM classifiers to obtain the average training accuracy without dividing them into training/test subsets.

In the braille recognition application, a robot arm (UR5, Universal Robots) equipped with the sensor was used to collect data. Each braille number (0–9) was captured by a portable board (Arduino Uno) based on a sliding‐touch process for 320 times, corresponding to 320 samples. Given that signals induced by braille bulges were expressed by small picks, a high‐pass filter was used to eliminate the effects caused by proximity. The same similarity‐matching algorithm was used to extract data samples to compose a dataset, with 280 samples for training and 40 for testing. To evaluate the effect of wearing variability, data were collected following the same setup and procedure. As a result, 400 samples (40 samples per Braille) were gathered as training data (denoted as dataset $S_{\mathrm{train}}$) and 200 samples (20 samples per Braille) as test data (denoted as dataset $S_{\mathrm{test}}^{1}$). Subsequently, the sensor was detached from the robotic finger and reinstalled, and new data were gathered, giving rise to 200 samples (20 samples per Braille) as new test data (denoted as dataset $S_{\mathrm{test}}^{2}$).

### ML Models

The SVM classifiers employed in the pre‐defined criteria were implemented in Python with a scikit‐learn package. A total of ten classifiers were constructed, with a regularization parameter ranging from 0.1 to 1.0. A one‐dimensional CNN model was developed in Python under the PyTorch framework. The CNN consisted of five convolution layers followed by batch normalization and rectified linear unit (ReLU) activation function each. The features encoded by the CNN were downsampled by an adaptive average pooling layer to a constant length before being fed into a multi‐layer perceptron classifier, which allowed for various‐length inputs. This model was trained using the adaptive moment estimation (Adam) optimizer for 100 epochs with a learning rate of 0.001 and a batch size of 128. It was subsequently used for inference on the test set and real‐time applications.

## Conflict of Interest

The authors declare no conflict of interest.

## Author Contributions

Y.L. and D.K. contributed equally to this work. Y.L., D.K., G.Y., and K.X. conceived the idea and designed the research. G.Y., G.P., H.L., and K.X. provided suggestions on device design and applications. Y.L. carried out the device fabrication, characterizations, and demonstrations. D.K. performed the data acquisition and analysis. R.W. carried out the manipulation of robotic arm in applications. Y.L., D.K., and K.X. wrote the manuscript. All the authors discussed the results and commented on the manuscript.

## Supporting information

Supporting Information

Supporting Information

Supplemental Video 1

Supplemental Video 2

## Data Availability

The data that support the findings of this study are available from the corresponding author upon reasonable request.
