# Supplementary material for: Machine Learning‐Enabled Tactile Sensor Design for Dynamic Touch Decoding
Source: Adv Sci (Weinh). 2023 Sep 22;10(32):2303949. doi: 10.1002/advs.202303949 (PMC10646241; doi:10.1002/advs.202303949)
Supplement: Supplementary file 1 — Supporting Information [file ADVS-10-2303949-s004.pdf]

ADVANCED SCIENCE
Open Access

Supporting Information

for *Adv. Sci.*, DOI 10.1002/advs.202303949

Machine Learning-Enabled Tactile Sensor Design for Dynamic Touch Decoding

Yuyao Lu, Depeng Kong, Geng Yang, Ruohan Wang, Gaoyang Pang, Huayu Luo, Huayong Yang and Kaichen Xu**

Supporting information

Machine Learning-Enabled Tactile Sensor Design for Dynamic Touch Decoding

Yuyao Lu[1†], Depeng Kong[1†], Geng Yang[1,2], Ruohan Wang[1], Gaoyang Pang[3], Huayu Luo[1], Huayong Yang[1], Kaichen Xu[1*]*

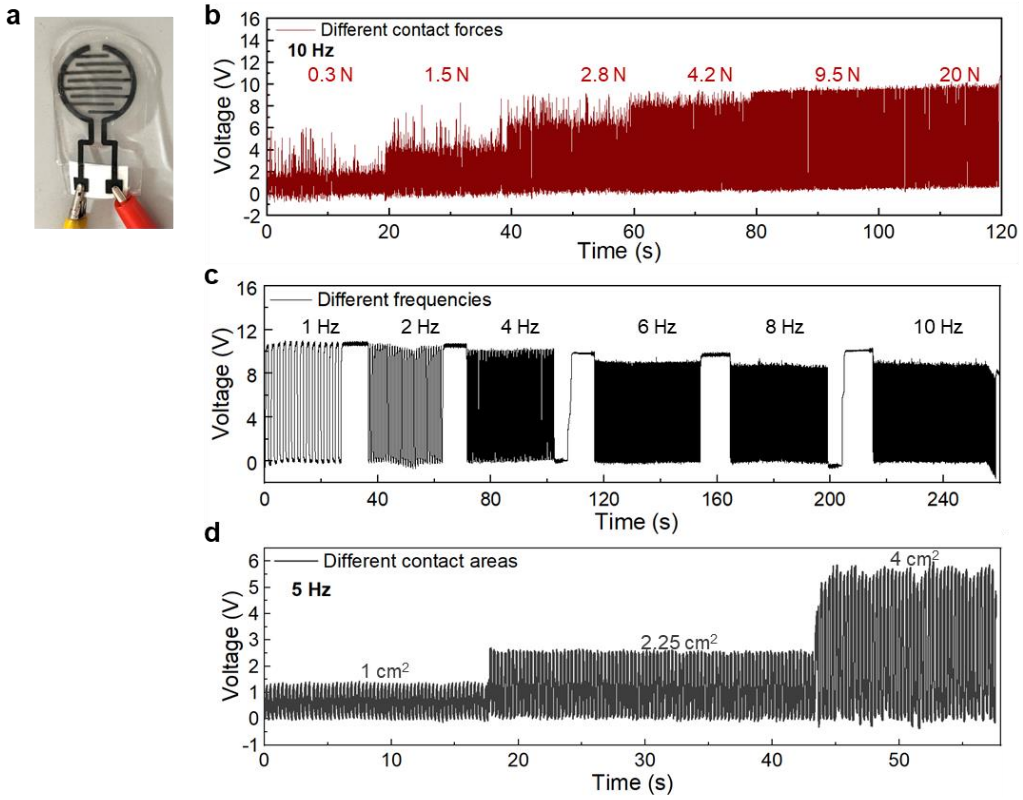

Figure S1. Characterizations of the TENG-based tactile sensor. a) Photo image of the sensor. Voltage output of the sensor under stimuli by different b) contact forces, c) frequencies and d) contact areas.

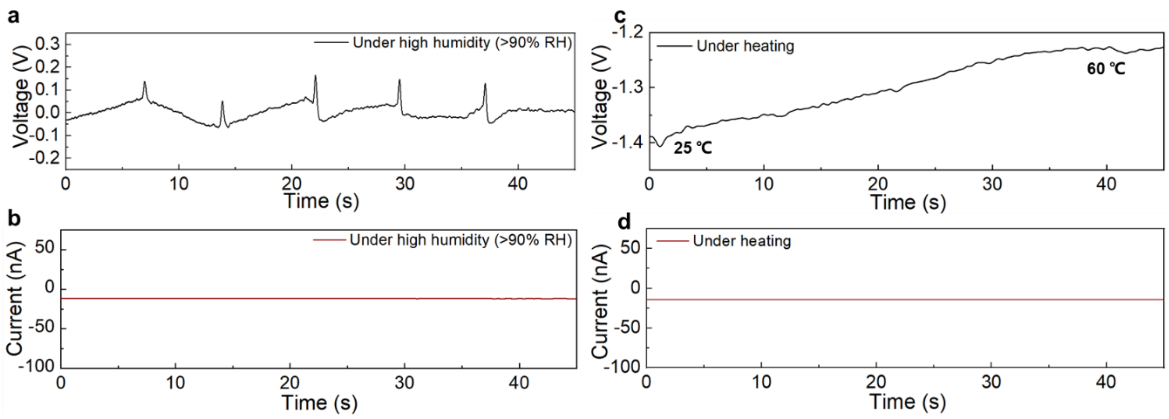

Figure S2. Investigation of humidity and temperature effects on sensor output.

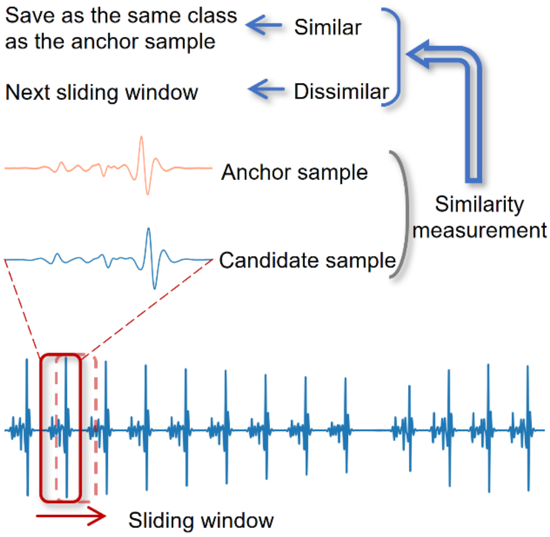

Figure S3. Diagram of the semi-automatic data annotation process.

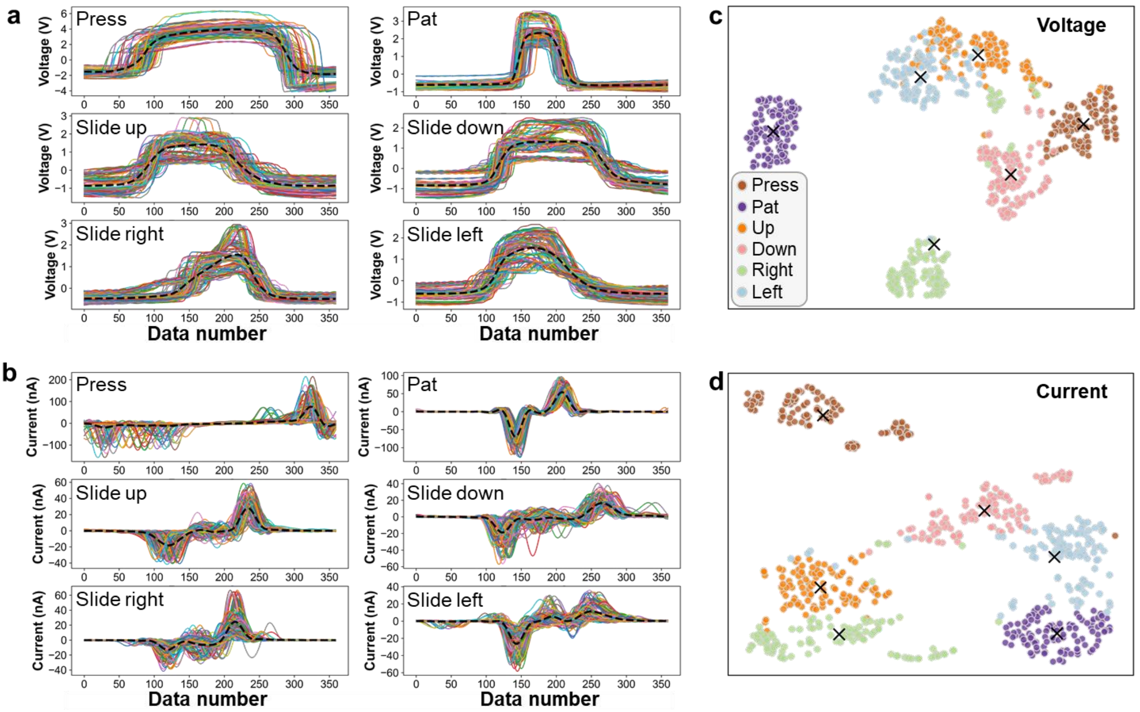

Figure S4. Comparison of types of the output signals in response to six touch modalities. Multiple cycle outputs in the form of a) voltage and b) current. Results of dimensionality reduction of data in the form of c) voltage and d) current.

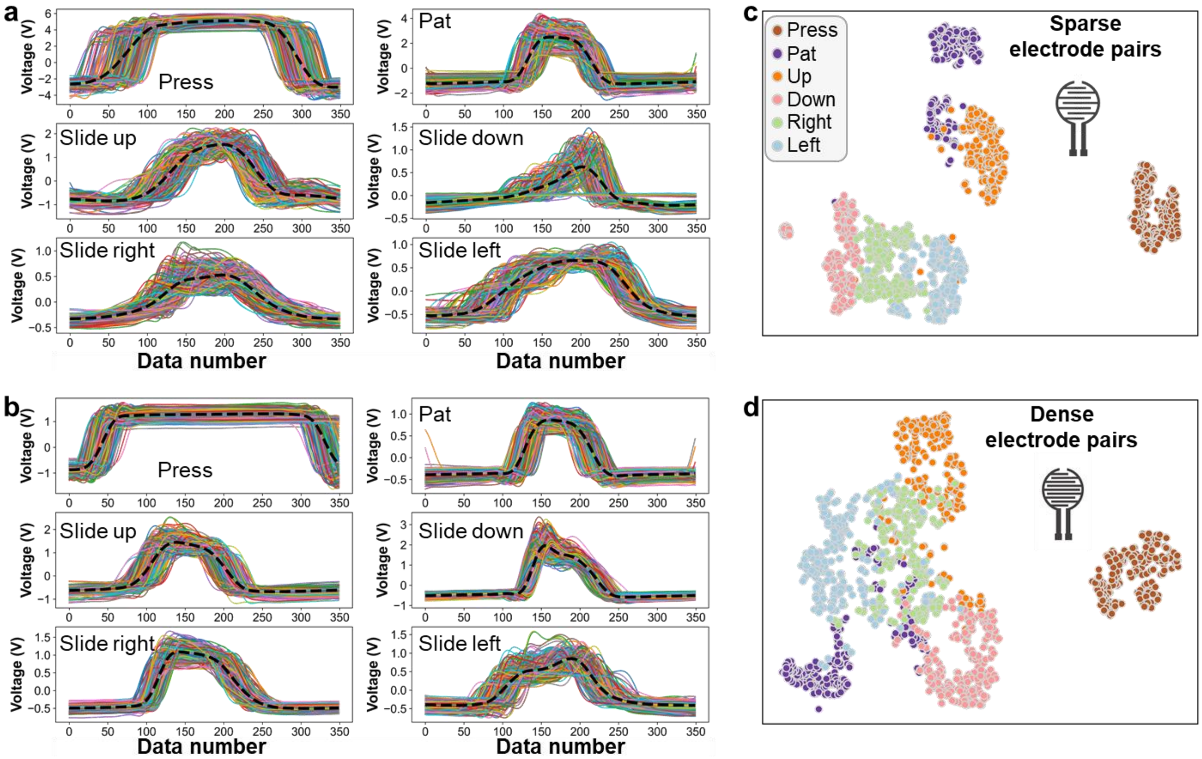

Figure S5. Comparison of sparse and dense arrangements of electrode pairs on the sensor output. Multiple cycle outputs of sensor with a) sparse electrode pairs and b) dense electrode pairs. Results of dimensionality reduction of data collected from sensors with c) sparse electrode pairs and d) dense electrode pairs.

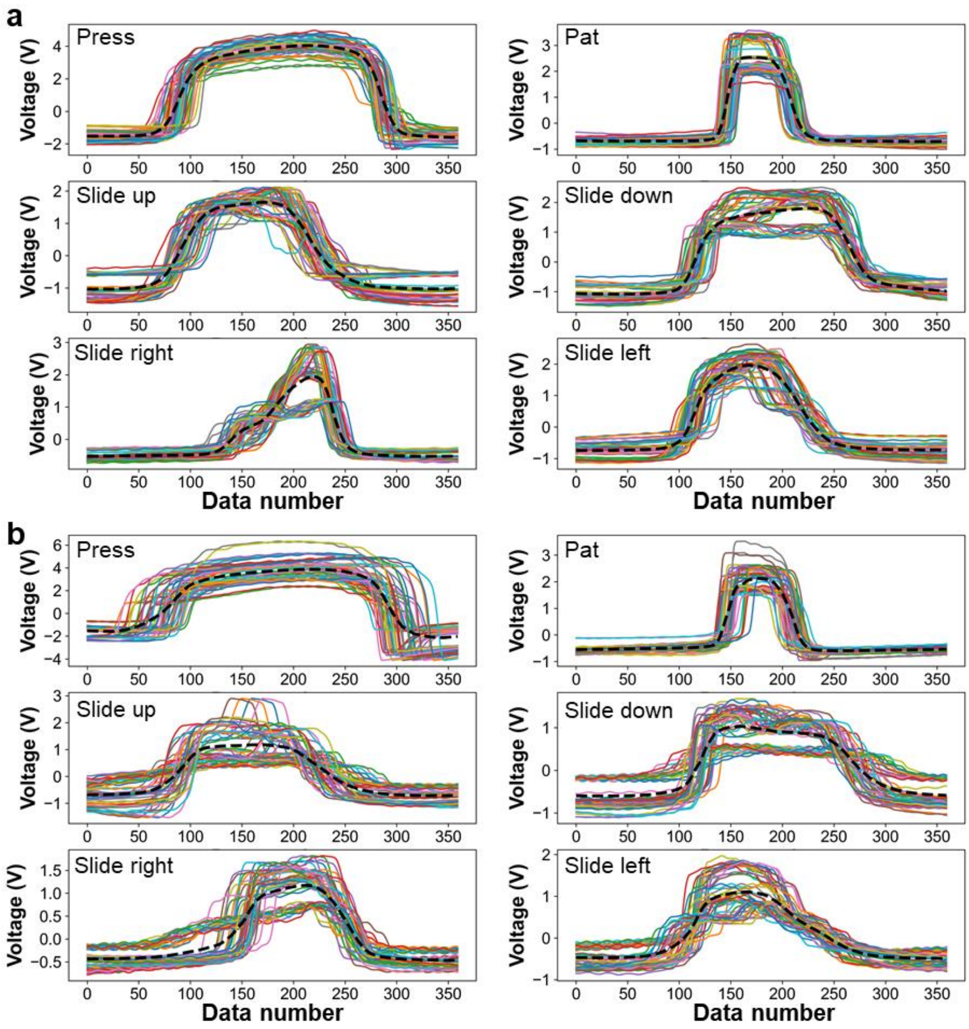

Figure S6. Comparison of different surface morphologies on the sensor output. Multiple cycle outputs of the data collected from sensor with a) fingerprint-like and b) grid-like shapes.

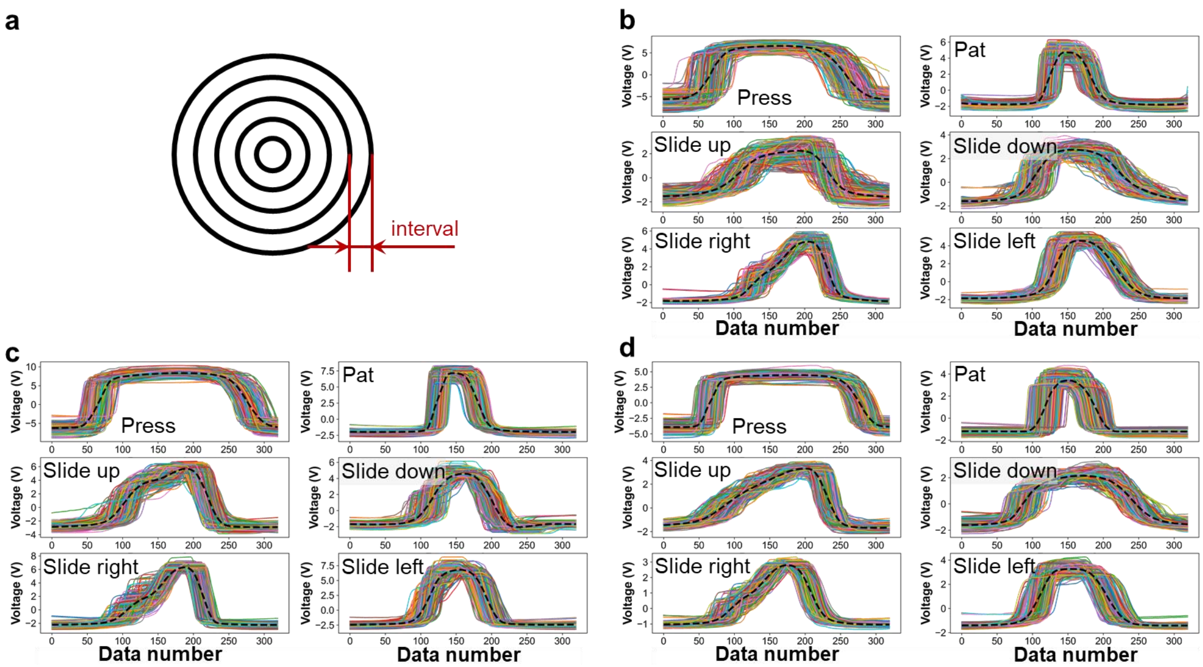

Figure S7. Comparison results of the tactile sensor with different groove intervals of the fingerprint-like microstructures. a) Schematic of the fingerprint-like shape. Multiple cycle outputs of the data collected from sensor with groove interval of b) 300 μm, c) 200 μm and d) 100 μm.

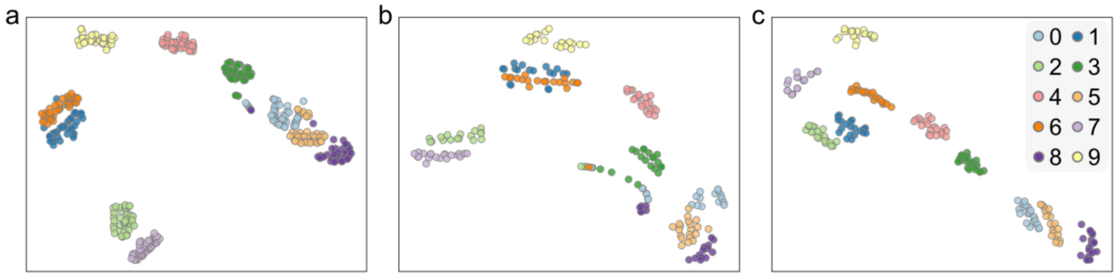

Figure S8. Results of dimensionality reduction by the t-SNE algorithm. a) Results of the training data (S_{train}) collected before the sensor's detachment. b) Results of the test data (S_{test}^{1}) collected before the sensor's detachment. c) Results of the test data (S_{test}^{2}) collected after the sensor's detachment and reinstallation.
