# Supplementary material for: Machine Learning‐Enabled Tactile Sensor Design for Dynamic Touch Decoding
Source: Adv Sci (Weinh). 2023 Sep 22;10(32):2303949. doi: 10.1002/advs.202303949 (PMC10646241; doi:10.1002/advs.202303949)
Supplement: Supplementary file 2 — Supporting Information [file ADVS-10-2303949-s003.pdf]

ADVANCED SCIENCE
Open Access

Supporting Information

for *Adv. Sci.*, DOI 10.1002/advs.202303949

Machine Learning-Enabled Tactile Sensor Design for Dynamic Touch Decoding

Yuyao Lu, Depeng Kong, Geng Yang, Ruohan Wang, Gaoyang Pang, Huayu Luo, Huayong Yang and Kaichen Xu**

Algorithm 1 Extract data samples of a particular class

Input: anchor sample: $a(t)$, time series: $X(t)$, sliding step: τ, similarity metric: $S(x_i, x_j)$, number of target samples: N.

Output: list C, which contains cut samples.

$L \leftarrow length[X(t)]$ ▷ length of time series $X(t)$
$W \leftarrow length[a(t)]$ ▷ length of anchor sample $a(t)$
$I \leftarrow (L - W)//\tau$ ▷ iterations cost for searching
$C \leftarrow []$ ▷ empty list for saving cut samples
$sim\_list \leftarrow 0_{[I \times 1]}$ ▷ array for saving calculated similarities
for $i = 1 \rightarrow I$ **do**
    $temp = X[i \times \tau : (i+1) \times \tau]$
    if $length(temp) == W$ **then**
        $sim \leftarrow S(a, temp)$
        $sim\_list[i] \leftarrow sim$
        append $temp$ to C
    end if
end for
rank C refer to sim_list in descending order.
$C \leftarrow C[0 : N]$
return C
